# Reversal of multidrug resistance in leukemia cells using a transferrin-modified nanomicelle encapsulating both doxorubicin and psoralen

**DOI:** 10.18632/aging.102992

**Published:** 2020-04-07

**Authors:** He-Wen Wang, Ke-Ling Ma, Hua Liu, Jia-Yun Zhou

**Affiliations:** 1 Department of Pediatrics, Rizhao People’s Hospital, Shandong, China

**Keywords:** Tf-nanomicelle, doxorubicin, psoralen, multidrug resistance, P-glycoprotein

## Abstract

To ameliorate multidrug resistance (MDR) observed in leukemia cells, nanomicelles modified by transferrin (Tf-M-DOX/PSO), coencapsulating doxorubicin (DOX) and psoralen (PSO), were designed, synthesized and tested in K562 and doxorubicin-resistant K562 (K562/DOX) cells. *In vitro* drug release kinetics for constructed nanomicelles were measured using high-performance liquid chromatography. Characterization of the produced nanomicelles was completed using transmission electron microscopy and dynamic light scattering. Uptake of the nanomicelles in K562 cells was investigated using both confocal microscopy and flow cytometry. Apoptosis levels as well as the expression of glycoprotein (P-gp) were analyzing by western blotting and flow cytometry. Cellular cytotoxicity resulting from the exposure of nanomicelles was evaluated using MTT assays. The nanomicelles all showed mild release of DOX in PBS solution. In K562/DOX cells, Tf-M-Dox/PSO exhibited higher uptake compared to the other nanomicelles observed. Furthermore, cellular cytotoxicity when exposed to Tf-M-Dox/PSO was 2.8 and 1.6-fold greater than observed in the unmodified DOX and Tf-nanomicelles loaded with DOX alone, respectively. Tf-M-Dox/PSO strongly increased apoptosis of K562/DOX cells. Finally, the reversal of the drug resistance when cells are exposed to Tf-M-DOX/PSO was associated with P-gp expression inhibition. The Tf-M-Dox/PSO nanomicelle showed a reversal of MDR, with enhanced cellular uptake and delivery release.

## INTRODUCTION

Leukemia is a common malignant hematopoietic stem cell disease classified as the 6^th^ most lethal cancer accounting for 4% of all cancer cases [1]. Currently, chemotherapy is the first treatment choice for this disease. In the clinic, multidrug resistance (MDR) is an obstacle to the satisfactory application of cancer chemotherapy. MDR can be induced by a single drug and shows tolerance to multiple drugs [2, 3]. Therefore, new approaches to reverse MDR are needed to improve chemotherapy as well as overall cancer treatment [4–6].

Over the last decade, several mechanisms that account for MDR have been reported [7–9]. Most studies agree with the view that MDR correlates with elevated levels of permeability glycoprotein (P-gp), an ATP-dependent efflux pump that efficiently decreases the concentration of administered medication introduced to the cell. Thus, various methods have been developed, such as therapeutic regimens in combination with inhibitors of P-gp and chemotherapeutic agents, to combat this issue. However, the use of traditional drug delivery is limited in clinical practice due to increased cytotoxicity [10–12].

Research related to small particle delivery systems, such as nanomicelles and liposomes, are promising approaches to improve cancer treatment as a platform for traditional chemotherapy drugs [13]. Nanomicelles and liposomes have potential to not only promote drug delivery into tumor tissues but also show promise in minimizing normal tissue exposure, ultimately improving drug efficacy and nonspecific cytotoxicity [14, 15]. In this study, we modified nanomicelles using transferrin conjunction, which is a commonly expressed iron-binding glycoprotein with a strong affinity for the Tf receptor (TfR). TfR is an essential mediator that induces endocytosis and is expressed frequently on tumor cells [16–18], including leukemia cells. This modification is applied to enhance the specificity of nanomicelles for the tumors [19, 20]. Furthermore, two drugs are encapsulated in the nanocarrier: doxorubicin and psoralen. Doxorubicin serves as the first-line drug in the treatment of leukemia and psoralen was recently reported to inhibit P-gp expression [21, 22].

In this study, the formulation of Tf-M-DOX/PSO was evaluated as a novel antileukemia drug in K562 cells as well as DOX-resistant K562 cells (K562/DOX). Here cellular toxicity, inhibition of expression of P-gp and cellular uptake were evaluated. We observed that Tf-M-DOX/PSO showed satisfactory performance *in vitro* for drug-related cellular uptake. In addition, novel Tf-M-DOX/PSO nanomicelles showed significant inhibition of cell proliferation regardless of DOX resistance. Finally, the reversal of drug resistance is correlated to the inhibition of P-gp expression. Therefore, the Tf-M-DOX/PSO formulation is a promising therapy and illustrates a novel approach to reverse MDR in leukemia patients.

## RESULTS

### Preparation of the nanomicelle complex

**Figure 1:**
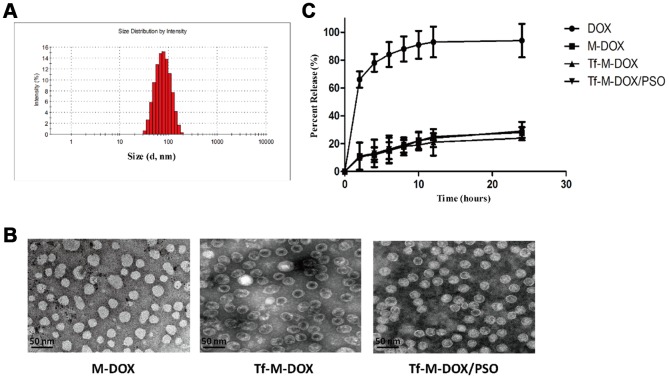
**Characterization of Tf-M-Dox/PSO nanomicelles.** (**A**) Particle size distribution of nanomicelles. (**B**) A Photographs of M/DOX, Tf-M-Dox and Tf-M-Dox/PSO were taken using transmission electron microscopy after staining with 1% uranyl acetate. Scale bar = 50 nm. (**C**) A time course of DOX release from various formulations at 37{degree sign}C in PBS is shown (n=3/group).

To facilitate the entrapment of DOX and PSO carrier loading, nanomicelles were synthesized by polycarbonate membrane extrusion. Drug loading of DOX and PSO was 19.2% and 17.4%, respectively whereas the incorporation efficiencies of DOX and PSO were 72.2% and 78.1%, respectively. The zeta potential of the nanomicelles was -10.9±1.3 mV and the mean diameter of a standard nanomicelle was 89.7 nm (Figure 1A). Tf-conjugated nanomicelle complexes were stable in PBS at 4°C for at least 2 months without detectable attenuation of DOX or PSO or a decrease in binding to Tf-expressing cells. Different nanomicelle complexes were photographed using a transmission electron microscope (Figure 1B). The further information of different combination drugs is listed in Supplementary Table 1.

### *In vitro* drug release

Qualified drug delivery systems require stable and sustained drug release. Considering that the pH of the blood is 7.35-7.45, the DOX release profile from the nanomicelles was determined in PBS by HPLC (Figure 1C). M-DOX, Tf-M-DOX and Tf-M-Dox/PSO showed mild release of DOX in PBS. In addition, the presence of Tf conjugation and PSO in Tf-M-Dox/PSO showed little effect on the rate of DOX release. Therefore, these data identified that the nanocarrier is a good platform for the controlled release of DOX.

### Cellular uptake

**Figure 2:**
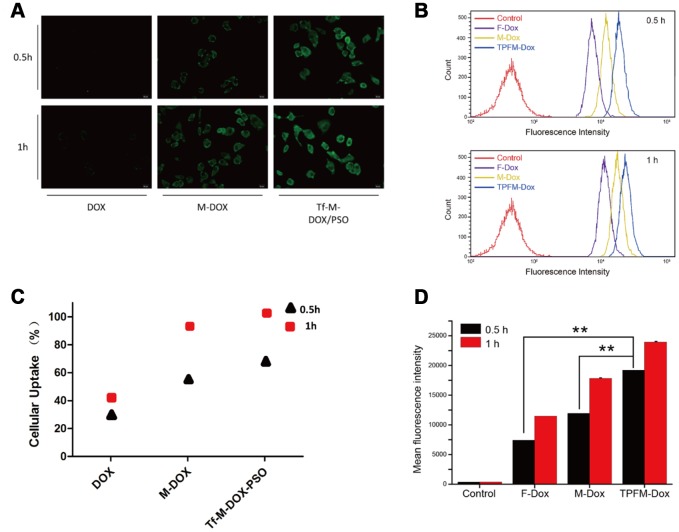
**Cellular uptake of DOX, M-DOX and Tf-M-DOX/PSO in K562/DOX cells.** (**A**) Cells were treated with DOX, M-DOX and Tf-M-DOX/PSO and then photographed by fluorescence microscopy. All samples were treated for 0.5 h or 1 h at 37°C. (**B**) Calculations of various micelle uptake by flow cytometry. Untreated cells were used as a negative control. (**C**) Positive percent of cells with fluorescence were illustrated. (**D**) Mean intensity of fluorescence in cells after 30 min or 60 min [of what?] (n=3/group). ** indicates P < 0.05.

We next tested K562/DOX cell cellular uptake of the different drugs on using a fluorescence microscope (Figure 2A). Each group of cells was treated with 10 μg/mL DOX, M-DOX and Tf-M-Dox/PSO for 30 or 60 min at 37°C. M-DOX was shown to have a higher uptake than the free 1DOX group, whereas Tf-M-Dox/PSO showed the strongest fluorescence among the groups. These data suggest that the Tf-M-Dox/PSO formulation enhances cellular uptake.

We also performed flow cytometry to investigate cellular uptake (Figure 2B). Cells treated with Tf-M-Dox/PSO showed fluorescence, which is in line with the fluorescence microscopy results. The cellular uptake in each group increased as the incubation time was extended from 30 min to 60 min (Figure 2C). Quantitative data from flow cytometry analysis are listed according to fluorescence intensity (Figure 2D). These results indicated that the mean intensity of Tf-M-Dox/PSO was 2.8-fold higher than M-DOX and 1.6-fold higher than DOX alone, in cells incubated for 0.5 h (both *p*<0.01). Therefore, this suggests that nano-carriers and Tf-conjugates can allow DOX to enter cells more quickly. The cellular uptake increased as incubation time was extended.

### Cytotoxicity of different DOX combinations in K562 cells and K562/DOX cells

**Figure 3:**
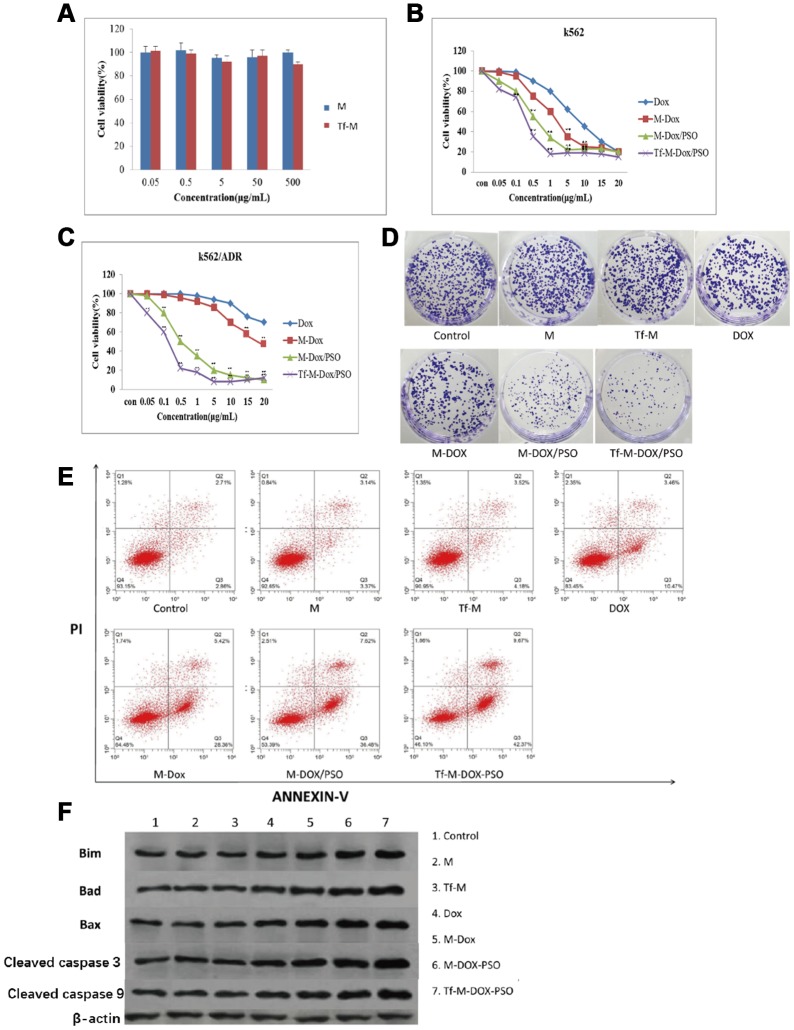
**Cytotoxicity of different combinations of DOX in K562 and K562/DOX cells.** (**A**) The cytotoxicity of both carriers was examined by MTT assays. (**B**) The antileukemia effect of four formulations of DOX in K562 cells was determined by MTT assays. (**C**, **D**) The antileukemia effects of different combinations of DOX in K562/DOX cells was examined by (**C**) MTT assays and (**D**) plate cloning experiments. of the effect on apoptosis caused by various delivery systems was examined by (**E**) flow cytometry and (**F**) western blot analysis. Data are shown as mean ± standard deviation (n=3), ** P < 0.05.

To study the effect of the two nanocarriers on K562 cells, an MTT assay was performed (Figure 3A). Both carriers showed little effect on the proliferation of K562 cells. The cytotoxicity of the various DOX formulations (DOX, M-DOX, M-DOX/PSO and Tf-M-DOX/PSO, all doxorubicin concentration at 10 μg/mL) was further examined in K562 cells (Figure 3B), Supplementary Table 2. These results showed that all formulation delivery systems have an inhibitory effect on cell proliferation in K562 cells. In addition, Tf-M-DOX/PSO showed the strongest inhibitory effect on K562 cells. These data suggest that doxorubicin in various formulations is capable of reducing the proliferation of K562 cells and that Tf-M-DOX/PSO has the strongest inhibitory effect on K562 cells.

Due to the emergence of MDR in patients with leukemia, many researchers have turned to K562/DOX cells as a tool to study MDR. K562/DOX is considered a valid surrogate model for clinical MDR leukemia. In contrast to K562 cells, the DOX and M-DOX groups exhibited a mild inhibitory effect on cell proliferation, while the M-DOX/PSO and Tf-M-DOX/PSO groups are still capable of reducing K562/DOX cell proliferation (Figure 3C), Supplementary Table 2. In line with results from MTT assays, the plate cloning experiment showed that only cells incubated with M-DOX-PSO or Tf-M-DOX/PSO exhibited obvious inhibitory effects on K562/DOX cells (all doxorubicin concentration at 15 μg/mL, Figure 3D). To further investigate the effect of the delivery system on cell apoptosis, flow cytometry was performed (Figure 3E). The results showed that cells treated with M, Tf-M or DOX showed no differences in cell apoptosis compared with the control group (doxorubicin=10 μg/mL). Slightly higher apoptosis was observed in the group treated with M-DOX. Importantly, greater levels of cell apoptosis were detected in K562/DOX cells incubated with M-DOX/PSO and Tf-M-DOX/PSO. Cell apoptosis was further verified by western blotting where the levels of proteins associated with apoptosis (BIM, Bad, Bax, caspase 3 and caspase 9) were higher in the cells treated with M-DOX/PSO and Tf-M-DOX/PSO (Figure 3F). Therefore, altogether, these data together indicate that DOX inhibits cell proliferation. In addition, the delivery system with PSO, especially Tf-M-DOX/PSO, is capable of reversing MDR in leukemia cells.

### Tf-M-DOX/PSO reversed MDR by inhibiting P-gp expression

**Figure 4:**
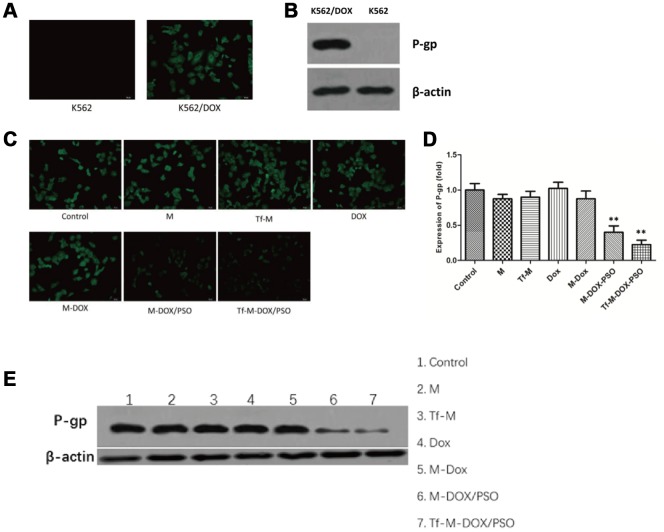
**Targeted delivery system inhibition of P-gp expression levels.** (**A**) The expression levels of P-gp in K562 cells and K562/DOX cells analyzed by fluorescence microscopy and (**B**) immunoblotting. The effects of various drug delivery system formulations on the delivery system were examined by (**C**) fluorescence microscopy (**D**) RT-PCR and (**E**) immunoblotting. Data are shown as mean ± standard deviations (n=3).

Our data thus far revealed that Tf-M-DOX/PSO inhibited cell proliferation in both K562 and K562/DOX cells. This may represent a novel combination for controlling MDR in leukemia. Next, our goal was to investigate the mechanism by which Tf-M-DOX/PSO inhibits MDR in leukemia. To verify the relationship between P-gp expression and MDR, the existence of P-gp was determined by fluorescence microscopy and western blotting (Figure 4A, 4B). The effect of the various DOX formulations (DOX, M-DOX, M-DOX/PSO and Tf-M-DOX/PSO, all doxorubicin concentration at 10 μg/mL) on P-gp expression was further examined in K562 cells by the fluorescence microscopy, western blotting and RT-PCR (Figure 4C–4E). These results showed that P-gp was highly expressed in K562/DOX cells. In addition, the delivery system containing psoralen inhibited P-gp expression where Tf-M-DOX/PSO showed the strongest inhibition on P-gp expression levels. Therefore, Tf-M-DOX/PSO has a powerful, lethal effect on both K562 and K562/DOX cells by inhibiting P-gp expression.

## DISCUSSION

The prognosis of leukemia is still unsatisfactory and better treatments are needed for this unmet clinical need. An analysis of cancer-related deaths reveals that leukemia serves as the most common cause of cancer in men under 40 years of age whereas female deaths from leukemia account for half of the cancer-related deaths. In addition, acute lymphocytic leukemia has become the most common cause of cancer among children under the age of 14 [1, 23]. Moreover, the incidence of chronic myeloid leukemia is 1-1.5 per 100,000 patients, one quarter of which an occurring relapse contributes a poor prognosis [24, 25]. Therefore, as there are limited treatments and poor prognosis for leukemia patients, MDR is an aspect of the disease that deserves more attention as well as further investigation. In this study, a novel Tf-conjugated nanomicelle coencapsulating DOX and PSO was prepared and examined in DOX-resistant K562 cells. Our data demonstrated that the nanocarrier with Tf coloaded DOX and PSO exhibits remarkable specificity for TfR-expressing cells and effectively overcomes drug resistance in K562/DOX cells.

Carrier size is a pivotal parameter for determining the physiological profiles of delivery efficiency. It has been indicated that nanoparticles with larger sizes will be enriched in the liver, spleen and other organs belonging to the reticuloendothelial system *in vivo*, while particles with a diameter of less than 100 nm tend to be taken up by the tumor [26, 27]. In addition, the transferrin receptor is highly expressed in various tumor cells, exhibiting the potential for targeted antitumor treatment [16, 19, 20]. The conjugation with transferrin is a common strategy to enhance the specificity of drugs [28]. In our study, Tf-M-DOX/PSO was designed and synthesized to improve antitumor performance. Our results showed that conjugation with Tf and the nanocarrier enhanced the antitumor ability of DOX compared to the control group. On this basis, the combination formulation remarkably inhibits proliferation and enhances apoptosis in K562 cells.

Recently, many researchers have investigated whether the application of various Tf-DOX formulations can affect P-gp-related drug resistance and further enhance the efficiency of antitumor therapy. Marzena et al. reported that the Tf-DOX formulation is a less P-gp-dependent substrate compared to free DOX [29]. In addition, several studies have shown that the combination of common antitumor drugs and nanosized carriers can be prepared with dendrimers, nanoparticles and micelles [30, 31]. These delivery systems, when employed with clinical drugs, can broaden the dose range of the drugs and enhance their accumulation in the tumor microenvironment. Similar to our study, Jun et al. demonstrated that transferrin-conjugated liposomes co-loading DOX and verapamil (Tf-L-DOX/VER) were prepared and exhibited high effectiveness in overcoming drug resistance in K562 cells [32].

Here, we demonstrated that the formulation of DOX as well as PSO co-loaded in nanomicelles remarkably reverses the expression of P-gp in K562/DOX cells [33–35]. A great deal of evidence has suggested the essential role of P-gp in the MDR of tumor cells, which has been reported to increase drug efflux and eventually lead to the impaired efficacy of chemotherapeutic drugs [36–39]. Even though there are no P-gp inhibitors that have been approved for clinical applications, big effort has been invested in identifying their potential role in alleviating MDR [40]. Several studies have indicated that psoralen, an effective constituent extracted from *Psoralea corylifolia*, could reduce MDR by regulating the activity of P-gp. It has been revealed that psoralen reverses MDR through inhibition of P-gp expression in MCF-7/ADR (human breast cancer cells) at pharmacologically feasible levels [21, 22]. However, to our knowledge, this is the first report of psoralen combined with DOX to inhibit drug-resistant leukemia cells. The drug efflux function of P-gp is essentially dependent on ATP hydrolysis [36, 37]. Psoralen is believed to inhibit the P-gp ATPase, thus reducing P-gp-mediated MDR. In addition, Psoralen has been reported to inhibit NF-kB activation, therefore influencing the extracellular matrix (ECM), a physiological activity that plays a pivotal role in the proliferation and metastasis of tumors, indicating that psoralen serves as a direct, negative regulator of tumor progression [41–43].

In conclusion, TfR-targeted coencapsulated DOX and PSO nanomicelles are a promising strategy for tumor specification and reversal of drug resistance in K562 cells. More investigation, especially proof in animal models, targeting the efficiency and toxicity of the new combination are needed in the future to identify whether this strategy can be implemented in leukemia patients.

## MATERIALS AND METHODS

### Preparation of nanomicelles

Nanomicelles coencapsulating doxorubicin, doxorubicin hydrochloride (Sigma-Aldrich, St Louis, MO, USA) were dissolved in 1 mL of absolute methanol, followed by the addition of triethylamine (DOX: triethylamine= 1:2, m/m). The addition of triethylamine converted the doxorubicin hydrochloride into the hydrophobic form of doxorubicin. This solution was mixed with PEG2000-DSPE (M.W.2000) dissolved in chloroform (Sigma-Aldrich) (DOX:nanomicelle = 1:5, m/m), stirred continuously at room temperature, and transferred to a flask. After the organic solvent was removed by a vacuum rotary evaporator, the drug coagulated to the lipid membrane in the bottom of the well. Next, 2 mL of PBS was added to the flask, followed by placing the flask in a 60°C water bath for 30 min. The solution was filtered using a 200 nm polycarbonate membrane using a nitrogen-driven Lipex^TM^ lipid extruder (Avanti Polar Lipids Inc).

To prepare nanomicelles coencapsulating doxorubicin and psoralen, psoralen (Sigma-Aldrich) was dissolved in 1 mL of absolute methanol and mixed with hydrophobic doxorubicin and PEG2000-DSPE. Nanomicelles composition contained PEG2000-DSPE/DOX/PSO at a mass ratio of 3:1:1. Preparation of these nanomicelles was similar to what was described above.

To conjugate Tf to nanomicelles, PEG2000-DSPE was replaced with PEG2000-DSPE and Tf-PEG2000-DSPE at a mass ratio of 4:1 (both PEG2000-DSPE and Tf-PEG2000-DSPE were purchased from RuixiBio, Xian, Shanxi, China).

### Cell culture

Chronic erythromyeloblastoid leukemia cells with amplified TfR (K562) and resistant to DOX (K562/DOX) were purchased from Fenghui Inc. K562/DOX originated from sensitive K562 cells by culturing them in the presence of gradual accumulations of DOX and maintaining the drug-resistant phenotype by exposure to 0.1 μM DOX. Cells were cultured in flasks and grown in RPMI 1640 supplemented with fetal bovine serum (FBS, 10%), penicillin and streptomycin (both 50 μg/mL). Cultures were grown at 37°C in a humidified incubator containing 5% CO_2_.

### Characterization of the nanocarrier

Tf-M-Dox/PSO nanomicelles were scattered in ultrapure water and the distribution of particle size and zeta potential were measured using a laser particle analyzer (Zetasizer 5000, Malvern Instruments, UK). To visualize Tf-M-Dox/PSO, Tf-M-Dox/PSO nanomicelles were dissolved in ultrapure water, immersed in carbon-coated copper mesh overnight, stained with 1% uranyl acetate and visualized using transmission electron microscopy (Tecnai G^2^ 20 S-TWIN, FEI, OR, USA). To determine drug loading and entrapment efficiencies, Tf-M-Dox/PSO was examined by HPLC. The HPLC chromatographic column used was a Syncronis C18 (250 mm ×4.6 mm, 5 μm). The mobile phase was a methyl alcohol-sodium acetate solution (pH=3.6, 65:35) at a flow rate of 1 mL/min. The solution was examined at a wavelength of 254 nm. The quantity of the sample injection was 30 μL. Lastly, the following formulas were used to calculate percentage drug loading and entrapment efficiency:

% drug loading= [(mass of drug in nanomicelle)/(mass of nanomicelle in the freeze-dried state)]×100%

% entrapment efficiency = [(mass of drug in nanomicelle)/(mass of drug added into the synthesis of the nanomicelle)] ×100%

### *In vitro* release of DOX from the nanocarrier

The rate of drug release from various formulations of the delivery system was examined by determining the retention of DOX in PBS. A 1 mL solution containing 50 μg of DOX in various formulations was added to a dialysis tube (Mw: 12000-14000). Then, the dialysis tube was closed, immersed in 30 mL of PBS (pH=7.4) at 37°C and placed in a shaker for cultivation at 100 r/min. One milliliter of solution from the dialysis tube was collected at 0, 2, 4, 6, 8, 10, 12, and 24 h and replaced with fresh PBS. The DOX in the solution was measured by HPLC as described above.

### Fluorescence assays

Fluorescence of the delivery system was performed to examine the uptake of DOX on leukemia cells. Briefly, DOX-sensitive and DOX-resistant K562 cells were incubated with various DOX concentrations for 0.5 and 1 h at 37°C. Then, cells were washed with PBS, followed by dissolution in ethanol and 20% SDS. The cells were then photographed by fluorescence microscopy (CytoViva Microscope System, Auburn, AL, USA).

To visualize the expression of P-gp levels, cells were seeded onto 6-well plates. After drug incubation, cells were washed three times with PBS and then fixed with 4% paraformaldehyde for 1 h at 37°C. Afterwards, these cells were incubated with an antibody recognizing P-gp (1:100, ab103477, Abcam, Cambridge, MA, USA) overnight at 4°C. After [how many?] cold PBS washes, cells were incubated with a fluorescence-labeled secondary antibody (1:500, ABclonal technology, Cambridge, MA, USA) for 1 h at 37°C and photographed.

### RT-PCR analyses

Cells treated with different delivery systems in the logarithmic growth phase with a confluence of 80-90% were collected for RT-PCR analyses. Total RNA was extracted from K562 or K562/DOX using TRIzol (Invitrogen, Carlsbad, CA, USA) according to manufacturer’s instructions. Next, extracted RNA was reverse transcribed into cDNA via SuperScript^TM^ II Reverse Transcriptase (Invitrogen). cDNA from each group was determined by RT-qPCR using SYBR Premix Ex Taq^TM^ (Takara, Beijing, China). GAPDH was used as an internal housekeeping control. Each sample was examined in triplicate and the cycle threshold values for specific gene were normalized to the endogenous control gene. The relative expression levels were determined according to the ΔΔCt method. Sequences of primers used in RT-PCR are as follows: GAPDH: forward primer, 5′-CATGAGAAGTATGACAACAGCCT-3′, reverse primer, 5′-AGTCCTTCCACGATACCAAAGT-3′; and P-gp: forward primer, 5′-AGCTCAAATGAGTGGAGGGC-3′, reverse primer, 5′-TGTAGTCCGACCTTTGCTCG-3′.

### MTT assay

The cytotoxicity of each drug combination including was evaluated using an MTT assay. K562 and K562/DOX cells were seeded in 96-well culture plates at a density of 7500 cells/well 1 d before the assay was performed. The culture medium was then replaced with medium containing different drug combinations, including free nanomicelles (M), transferrin-modified nanomicelles (Tf-M), free DOX, nonmodified nanomicelles with DOX (M-Dox), nonmodified nanomicelles with DOX and PSO (M-Dox/PSO) and transferrin-modified nanomicelles with DOX and PSO (Tf-M-Dox/PSO), with the doxorubicin concentration at 10 μg/mL. Following 6 h of incubation at 37°C, each group of cells was washed three times with PBS and cultured in fresh medium for another 3 d. Next, 20 μL of MTT reagent (5 mg/mL, Sigma-Aldrich) was added to each well and the cells were incubated for an additional 4 h at 37°C. Culture medium was replaced with DMSO to lyse the blue formazan crystals generated from live cells. Finally, cell viability of each group was determined by measuring the absorbance using a microtiter plate reader at 570 nm (Bio-Rad, Hercules, CA, USA).

### Flow cytometry

To investigate the effect of various formulations of DOX on cell apoptosis, an Annexin V apoptosis detection kit (BD, Franklin Lakes, NJ, USA) was utilized. Briefly, cells exposed to the various formulation of DOX (10 μg/mL) for 1h were washed three times with PBS and resuspended in 100 μL of 1× binding buffer with 5 μL of Annexin V-FITC. Then, the cells were incubated at 4°C in the dark for 5 min before analyzing cell apoptosis by flow cytometry.

To quantitatively measure the amount of DOX uptake, K562/DOX cells were incubated for 0.5 h or 1 h at 37°C with DOX, M-DOX or Tf-M-Dox/PSO. Then, cells were washed three times with PBS and examined by flow cytometry.

### Immunoblotting

To investigate the expression of P-gp levels in K562 cells treated with various formulations of DOX (10 μg/mL), western blotting was performed. Briefly, total protein lysates were collected in a lysis solution containing 1% PMSF. And total protein concentration was measured using a BCA protein assay kit (CWBIO, Beijing, China). Equal amounts of protein from each sample were separated using 10% SDS-PAGE gels before transferring to a polyvinylidene difluoride membrane. Membranes were blocked in nonfat milk and then incubated with primary antibodies against P-gp and β-actin (Santa Cruz Biotech, #sc-47778) overnight at 4°C. The next day, blots were incubated with a secondary antibody at 37°C for 2 h. Bands were visualized using ECL according to the manufacturer’s protocol.

Antibodies against Bim (ab32158), Bad (ab32445), Bax (ab32503), caspase 3 (ab197202) and caspase 9 (ab219590) were purchased from Abcam. Equal loading between samples was determined analyzed by β-actin expression levels.

### Plate cloning

K562/ADR cells were cultured in 10 cm dishes and treated with 15 μg/mL of different drug combinations including free nanomicelles (M), transferrin-modified nanomicelles (Tf-M), free DOX, nonmodified nanomicelles with DOX (M-Dox), nonmodified nanomicelles with DOX and PSO (M-Dox/PSO) and transferrin-modified nanomicelles with DOX and PSO (Tf-M-Dox/PSO) for 24 h. Cells were cultured inRPMI1640 medium containing 10% FBS for 15 d. After being fixed and stained, each group of clones (more than 50 cells were considered as a clone) were calculated. The inhibition rates of different drugs were counted as follows: Clone formation rate (%) = average number of clones/number of seeded cells×100; and Inhibition rate (%) = (1-clones number of the experimental group/clones number of the control group) × 100.

### Statistics

Data are listed as the mean ± standard deviation (SD) and were calculated using SPSS V 17.0. Student’s t-test was utilized to compare the means of two groups. One-way ANOVA was utilized to compare the means of multiple groups, followed by Dunnett’s t-test. *P<*0.05 was considered significant.

## Supplementary Material

Supplementary Tables
