# Supplementary material for: Reversal of multidrug resistance in leukemia cells using a transferrin-modified nanomicelle encapsulating both doxorubicin and psoralen
Source: Aging (Albany NY). 2020 Apr 7;12(7):6018–29. doi: 10.18632/aging.102992 (PMC7185111; doi:10.18632/aging.102992)
Supplement: Supplementary Tables [file aging-12-102992-s001..pdf]

SUPPLEMENTARY TABLES

Supplementary Table 1. Characterization of different drug combination. Results are expressed as mean ± SD (n = 3–5).

	Particle Size (nm)	Drug loadings (DOX)%	% Entrapment
M-Dox	78.1±0.84	22.4	75.3
Tf-M-Dox	83.1±0.67	20.4	68.9
Tf-M-Dox/PSO	89.7±0.73	19.2	72.2

Supplementary Table 2. Cytotoxicity of various DOX formulation on K562 and K562/ DOX cells.

	IC50 (uM)	
	**K562**	**K562/DOX**
Dox	7.71±1.42	27.1±4.5
M-Dox	6.67±1.04	14.2±2.3
M-Dox/PSO	5.52±0.81	6.98±1.03
Tf-M-Dox/PSO	1.39±0.37	3.26±0.42
